# Supplementary material for: Assessment of adherence to the World Health Organization’s prescribing indicators at the family medicine clinic of a quaternary facility
Source: PLoS One. 2026 Feb 12;21(2):e0341088. doi: 10.1371/journal.pone.0341088 (PMC12900328; doi:10.1371/journal.pone.0341088)
Supplement: Attachment — Submitted filename: Response to Reviewers.docx [file pone.0341088.s001.docx]

Nana Ama Buadiba Osei

University of Ghana Medical Centre

16/11/2024

The Academic Editor

PLOS ONE

Rebuttal Letter for Manuscript ID [PONE-D-24-26584]

Dear Fatima,

Thank you and the reviewers for providing insightful feedback on my manuscript, "Assessment of adherence to the World Health Organization’s Prescribing Indicators at the Family Medicine Clinic of a Quaternary Facility." I have carefully considered each comment and have revised the manuscript accordingly. Below, I provide detailed responses to each point raised.

**Response to Reviewer #1**

1. Comment 1: Study design - Is it a cross-sectional design? Prescription records of the past have been studied, so it should be a retrospective record-based design.
a) Response 1: I agree that the study design requires further clarification. The study involved two components: a retrospective review of prescription data from patient medical records to assess WHO core prescribing indicators at UGMC from January 2022 to May 2023, and interviews with prescribers conducted during the data collection period in August 2023. These interviews aimed to gather additional insights into prescribing practices. The study design has been updated to reflect this dual approach in the "Methods" section.
2. Comment 2: Random selection of the samples needs to be elaborated. 50 prescriptions every month for 17 months adds up to 850 prescriptions, but 600 only have been studied. How was this reduction made?
b) Response 2: I acknowledge the need for clarity. The WHO guideline that this study followed recommends at least 600 encounters for such studies (Ofori-Asenso, 2016; World Health Organization, 1993). Following the extraction of the 23,690 prescriptions, the data was exported to the STATA software where a command was issued to randomly select 600 prescriptions systematically from the total number of prescriptions. This helped ensure manageable data analysis while maintaining representativeness. Initially, the approach involved selecting 50 prescriptions monthly but this was later modified. Its inclusion in the write-up was a typographical error, which has been corrected in the revised manuscript.
3. Comment 3: Is the IRDP a validated index? The WHO recommended optimal values for some indicators are not absolute numbers but "below a cut-off value." How can the index be calculated considering the existence of a wide range of numbers below the cut-off?
c) Response 3: The IRDP was adapted from established methodologies used in similar studies and is rooted in the mathematical model developed by Zhang and Zhi (1995), which has been widely utilized to evaluate rational drug use (RUM). This model assigns an optimal index of 1 for each prescribing indicator, where values closer to 1 indicate more rational prescribing. While the WHO-recommended values for some indicators are presented as thresholds (e.g., "below a cut-off value"), the IRDP provides a standardized approach to quantify deviations from these recommendations, enabling consistent comparisons.
4. Comment 4: Table 3 could be avoided by just adding one more row to Table 2 with 'Overall IRDP' and mentioning the optimal IRDP in the text.
d) Response 4: Table 3 has been removed, and the "Overall IRDP" has been added as a row in Table 2. The optimal IRDP has been mentioned in the text for clarity.
5. Comment 5: In the results, many tables with regression analysis for the association of demographic factors have been reported. However, these findings are not reflected in the conclusion.
e) Response 5: The conclusions have been revised to incorporate the key findings of the regression analyses, particularly the associations between demographic factors and prescribing patterns.
6. Comment 6: Though this is a quaternary facility, there is no mention of the availability of the prescribed drugs in the facility. Is it a government or private facility?
f) Response 6: Thank you for highlighting this omission. The manuscript now specifies that the study was conducted in a quaternary, government-funded facility, and the availability of prescribed drugs has been briefly discussed in the "Discussion" section.

**Response to Reviewer #2**

7. Comment: Include a few lines in the Discussion on reasons for less than optimum values for some prescribing indicators (e.g., generic prescribing and prescribing from the EML or hospital formulary) and what measures were taken or planned to be taken to improve these.
g) Response: I have expanded the "Discussion" section to explore reasons for less-than-optimal values under generic prescribing and prescribing from the EML including the inactivity of the Drugs and Therapeutics Committee as well as the absence of a medicines formulary in the facility. I have also indicated some measures that can be taken to improve these values including the reinstatement of the DTC and providing them with the resources needed to work as well as the development and dissemination of a hospital formulary to prescribers.
8. Analysis of Results

The results for the inferential analysis of the regression models have been modified.

**Association between demographic characteristics and number of drugs prescribed**: A Poisson regression model was carried out and the prevalence rate was interpreted because when using Poisson regression for cross-sectional data, the effect estimate is the prevalence rate.

Logistic regression of demographic characteristics and the presence of an antibiotic in a prescription: The interpretation has been modified considering the reference point for Odds Ratio, and any other ratio, is 1.

Zero-inflated Poisson regression of demographic characteristics and number of drugs prescribed from the Essential Medicines List of Ghana: Interpretation has been modified because when using Poisson regression for cross-sectional data, the effect estimate is prevalence rate.

9. Changes to the reference list
h) More current references have been included in the Discussion under polypharmacy.

Thank you for your time and consideration. I look forward to your feedback on the revised manuscript.

Sincerely,

Nana Ama Buadiba Osei
