# Supplementary material for: Assessment of adherence to the World Health Organization’s prescribing indicators at the family medicine clinic of a quaternary facility
Source: PLoS One. 2026 Feb 12;21(2):e0341088. doi: 10.1371/journal.pone.0341088 (PMC12900328; doi:10.1371/journal.pone.0341088)
Supplement: Attachment — Submitted filename: Response to Reviewers V2.docx [file pone.0341088.s002.docx]

Nana Ama Buadiba Osei

University of Ghana Medical Centre

16/11/2024

The Academic Editor

PLOS ONE

Rebuttal Letter for Manuscript ID [PONE-D-24-26584R1]

Dear Dr. Adedia,

Thank you and the reviewers for providing insightful feedback on my manuscript, "Assessment of adherence to the World Health Organization’s Prescribing Indicators at the Family Medicine Clinic of a Quaternary Facility." I have carefully considered each comment and have revised the manuscript accordingly. Below, I provide detailed responses to each point raised.

**Response to Reviewer #3**

1. Comment 1: Any reason why patients at the out-patient pharmacy were being prescribed injections would be very much appreciated.
a) Response 1: The Out Patient department has a 24-hour detention area for patient observation, such patients are administered stat doses of intravenous medications and are subsequently discharged or admitted based on the physician’s assessment.
2. Comment 2: Your citation of studies is not correct. For example, Mahali (2012) and Baba and Salifu (2019) are totally out of place. There is another one on page 6, line 128.
b) Response 2: References have been corrected accordingly
3. Comment 3: Table 1 is too scanty. I was expecting to see more variables here. For example, you could show a categorical variable of number of drugs per prescription, that is < 1 (0), 1-2, 3-4, >4, and so on.
c) Response 3: The data obtained was analyzed in direct relation to the objectives of study which focused on the average number of drugs prescribed per visit not per prescription
4. Comment 4: If you have 23,690 records and the minimum required is 600, why did you not go for more to be extra sure of the sure of the study power?
d) Response 4: Owing to a lack of resources and time constraints the number of records assessed was limited to the minimum required by the WHO. A larger sample size will be employed in follow-up studies
5. Comment 5: Page 7, line 148, you mentioned that patient identities were either removed of anonymized. You need to be consistent here. What was actually done to patient identities?
e) Response 5: Patient identifiers were anonymized during the extraction process and before analysis, they were deleted completely. This will be clearly indicated in the manuscript.
6. Comment 6: Kindly explain the rationale behind the use of zero-inflated Poisson regression in your study. What is the relationship between the mean number of drugs prescribed in this study and the variance?
f) Response 6: The zero-inflated Poisson regression was used for the “number of injectables” and “antibiotics found on a prescription”. A prescription can have at least one medication but this medication may neither be an injectable nor an antibiotic. This increased the number of zeros (0) data points found under these variables hence the use of the zero-inflated Poisson regression. The variance was not reported on in this study with respect to the number of drugs prescribed
7. Comment 7: What is the meaning of “mean age of the patient was 43.82+17.88 years? The mean normally goes with the standard deviation. Why do you have inter-quartile range (IQR) here? The IQR normally goes with the median. These are not on Table 1, however you have referred to Table 1. Why is that so?

Response 7: This statement should read “mean age of the patient was 43.82(SD =17.88 years) and the inter-quartile range has been taken out. The reference to table one was for all other statements made in that paragraph. Reference has been shifted up to reflect that

8. Comment 8: It will be better to have “observed values” and “optimal values” for Table 2 to help reviewers calculate the index value as specified in your methods.

Response 8: The values for observed and optimal are written in the text following the recommendation of one of the reviewers to reduce the number of tables presented.

9. Comment 9: The p-values for Age as indicated in both Tables 5 and 6 look too high compared to the 95% confidence intervals indicated. Kindly check your statistical values again.

Response 9: Below is the direct output from stata after the raw data was analyzed.

10. Comment 10: Are you IRR values coefficients or real rate ratios?

Response 10: They are the coefficients

11. Comment 11: On page 21, line number 402, you stated that “for those with comorbidities the number of drugs was 0.346 times higher… Are you saying that those with comorbidities had less drugs compared to those without comorbidities? There is a similar statement on line 412. On page 9 you stated that patients with comorbidities have a 52.2% lower prevalence rate of the total number of medicines prescribed compared to those without comorbidities. However, your abstract says that patients without comorbidities were 65.4% less likely to have polypharmacy. These are quite confusing. It well established that patients with comorbidities have more prescribed medicines because more diseases that are not related may be treated. You need to look at your discussion again and explain your statistical analysis well. These seem to be a few discrepancies here and there.

Response 12: From the analysis done patients with comorbidities have a 52.2% lower prevalence rate of polypharmacy compared to those without comorbidities. The text has been refined to clarify this.

**Response to Reviewer #4**

1. Comment (Abstract): Provide adjusted odds ratio along with 95% CI for significant independent variables. I suggest the authors to include a concise recommendation in the conclusion section.

Response: Adjusted odds ratios have been included and the following recommendations have been made in the document

2. Comments (Methods and Results)
   a. The study design is cross-sectional. I suggest the authors to omit ‘retrospective’ in the study design. Simply indicate that data were collected retrospectively in the ‘Data collection tool and approach’ section. This is because retrospective study design is one of the longitudinal study designs in which the dependent variable and independent variables are measured at different points in time.

Response: Corrections have been made

b. Massive English editing required. Coherence and readability of the manuscript need improvement.

Response: The appropriate corrections have been made

c. Clearly state the selection criteria of the study setting over other clinics in Ghana.

Response: This study was carried out at UGMC; a newly established hospital, to provide baseline information beneficial in identifying quality improvement areas for subsequent interventions to improve prescribing practices.

d. For ease of understanding and readability, I suggest the authors to segregate the method section into sub-sections such as study design and setting, target population, sample size determination, data collection tool and approach, variables, data quality control, ethical consideration, and statistical analysis.

Response: The various subheadings have been included

e. Provide an operational definition for polypharmacy and rational medicine use.

Response: Rational medicine use encompasses all the dependent variables being studied namely; Polypharmacy, Generic name-based prescribing, Antibiotics prescribed, Injections prescribed, Adherence to EML prescribing and polypharmacy is defined as the number of medications prescribed per patient encounter.

f. Did you conduct a face and content validity, and pre-test for the questionnaire that was used in interviewing the prescribers?

Response: The questionnaire was pre-tested among prescribers from the anesthesia, internal medicine and surgery outpatient clinics at UGMC and then reviewed where necessary based on the responses received.

g. Citation for index of rational drug prescribing is incorrect [Page 6; Line 128].

Response: Citation has been modified according to PLOS requirements

h. Did your study conform to the principles outlined in the Declaration of Helsinki?

Response: Yes

i. State the optimal values for the WHO prescribing indicators in a table format.

Response: The values for observed and optimal are written in the text following the recommendation of one of the reviewers to reduce the number of tables presented and present the optimal values as presented in the manuscript

j. How was the 600 prescriptions selected from the total 23,690?

Response: The WHO guideline that this study followed recommends at least 600 encounters for such studies (Ofori-Asenso, 2016; World Health Organization, 1993). Following the extraction of the 23,690 prescriptions, the data was exported to the STATA software where a command was issued to randomly select 600 prescriptions systematically from the total number of prescriptions. This helped ensure manageable data analysis while maintaining representativeness.

k. Explain in detail the statistical analysis used in this study.

Response: The dependent variables were all count variables thus the use of the Poisson regression model to establish an association. However, the zero-inflated Poisson regression was used for the number of injectables or antibiotics found on a prescription. A prescription can have at least one medication but this medication may neither be an injectable nor an antibiotic. This increased the number zeros (0) values found under these variables hence the use of the zero-inflated Poisson regression. Logistic regression was carried out to establish associations between dependent variables that were categorical variables such as the association between demographic data and whether there was an antibiotic in the prescription

l. What is your inclusion criteria and sampling technique utilized for recruiting the prescribers?

Response: The inclusion criteria consisted of all physicians who were actively involved in prescribing medications to patients at the family medicine clinic during the period of data collection. All prescribers who were on duty during the study period were sampled due to the small sample size.

m. The knowledge and training of prescribers towards rational use of medicines, contact of prescribers with pharmaceutical sales representatives, and work-related factors are not in line with the objectives of the study. It cannot be representative to the views of the ordinary prescribers.

Response: The database used did not link prescribers to their prescriptions, which was a limitation of the study. This connection would only be possible if the study were conducted in real time. However, through discussions with prescribers, I gained valuable insights into their collective behaviors and the factors influencing their prescribing patterns. Although the prescriber data was not directly linked to individual prescriptions, it provided important context to the study’s outcomes. Future studies could address this limitation by collecting data in real time for a more detailed analysis.

n. Explain in detail the number of items included in the questionnaire. State the number of open-ended and closed-ended questions.

Response: 20 items were included in the questionnaire. Below is an explanation of the questions in the questionnaire.

**Section A: Demographic details of Respondents**

Sex: Captures the respondent's gender (Closed-ended).

Age: Groups respondents into predefined age ranges (Closed-ended).

Profession: Identifies the respondent’s professional designation, with an option to specify others (Closed-ended with "Other" option).

Years of Practice: Assesses prescribers' experience in years (Closed-ended).

Understanding of Rational Use of Medicines (RUM): Evaluates whether respondents know the definition of RUM (Closed-ended).

Parameters of RUM Standards: Tests knowledge of RUM standards (Closed-ended with an "Any other" option).

**Section B: Training on RUM**

Training on RUM: Asks if the respondent has undergone training on RUM (Closed-ended).

Last Training Date: Determines when the training occurred (Open-ended).

Training Benefits: Assesses the perceived usefulness of the training (Closed-ended).

Training Frequency: Inquires about the ideal training schedule (Closed-ended with "Other" option).

**Section C: Contact with Pharmaceutical Sales Representatives**

Frequency of Visits: Captures how often prescribers interact with sales reps (Closed-ended).

Interaction Locations: Identifies where these interactions occur (Closed-ended).

Interaction Duration: Estimates the typical duration of interactions (Closed-ended).

**Section D: Work-Related Factors That Affect the Rational Use of Medicines**

Guideline Availability: Inquires if prescribers have access to guidelines (Closed-ended).

Guideline Reference Frequency: Determines how often guidelines are used (Closed-ended).

Information Sources: Assesses reliance on various information sources for prescribing decisions (Closed-ended).

Functional Drug and Therapeutics Committee: Verifies the existence of a relevant committee at the facility (Closed-ended).

Perception of Representatives and Committees: Seeks agreement on the value of pharmaceutical representatives and therapeutic committees using a Likert scale (Closed-ended).

**Section E: Reasons for Non-Adherence to RUM**

Factors for Non-Adherence: Explores reasons for not fully adhering to RUM standards (Closed-ended).

Total Open-Ended Questions: 2

Total Closed-Ended Questions: 18.

3. Comments (Discussion)
   a. Reason in the difference of results from the current study and other similar studies were not stated. Moreover, provide policy implications for the various findings in your study.

Response: Justification for the variation in findings has been provided

b. Explain in detail why antibiotic prescription in Ghana was consistent with the WHO standard. Such sort of information will provide an insight and lesson to countries with similar socio-economic status.

Response: In UGMC there is an active antimicrobial stewardship committee that develops interventions to regulate the use of antibiotics according to WHO standards. This has been stated in the discussion.

c. Avoid detail figures (for instance 95% CI) in the discussion section and take more emphasis on interpreting the results while providing explanations and policy implications for the findings.

Response: Confidence interval values have been removed from the values in the discussion.

d. State the limitations of the study.

Response: On the other hand, a focus on just one institution as well as a small sample size relative to the overall population may limit the generalizability and representativeness of the findings from this study.

e. Provide recommendations along with the conclusion of the study.

Response: The following recommendations have been added to the conclusion; Rational drug prescribing may be enhanced through training, guidelines, EML distribution, drug and therapeutics committee support and integrated Clinical Decision Support Systems (CDSS). Additionally, enforcing DTCs in healthcare institutions, and expanding research to include patient and healthcare provider perspectives would also greatly improve rational use of medicines.

Thank you for your time and consideration. I look forward to your feedback on the revised manuscript.

Sincerely,

Nana Ama Buadiba Osei
