# Supplementary material for: Assessment of adherence to the World Health Organization’s prescribing indicators at the family medicine clinic of a quaternary facility
Source: PLoS One. 2026 Feb 12;21(2):e0341088. doi: 10.1371/journal.pone.0341088 (PMC12900328; doi:10.1371/journal.pone.0341088)
Supplement: Attachment — Submitted filename: Response to Reviewers V4.docx [file pone.0341088.s003.docx]

Nana Ama Buadiba Osei

University of Ghana Medical Centre

10/07/2025

The Academic Editor

PLOS ONE

Rebuttal Letter for Manuscript ID [PONE-D-24-26584R1]

Dear Dr. Adedia,

Thank you and the reviewers for providing insightful feedback on my manuscript, "Assessment of adherence to the World Health Organization’s Prescribing Indicators at the Family Medicine Clinic of a Quaternary Facility." I have carefully considered each comment and have revised the manuscript accordingly. Below, I provide detailed responses to each point raised.

**Response to Editor’s Comments**

Comment: The headings under discussion should be removed. The topic-specific discussions could be put in a paragraph.

Response: The subheadings in the discussion have been removed

**Response to Reviewer #3**

1. Comment 1: The response to comment 3 is not fully acceptable. First of all, the average number of drugs per prescription is in itself a benchmark of the WHO instrument. Secondly, a reviewer may request the researcher to add further information for the sake of clarity.

Response: This is well noted. For this study, we wanted to focus on the WHO indicators as this is a baseline study, and future studies will explore in detail the number of prescriptions.

2. Comment 4, the researcher mentioned lack of resources and time constraints as reasons for not being able to use more information from the database. It is fine if you decide to use the minimum data size, but lack of resources and time constraints are not good reasons if you want to do good research. Besides, the research involved secondary data extracted from an existing database.

Response: We acknowledge the disadvantage of a smaller dataset and will rectify that inn future studies.

3. Comment 7, your abstract still has, “The mean number of 33 prescribed medications was 1.4 (n=840)…” instead of mean and standard deviation. There is a very good reason why it is recommended that the standard deviation should be quoted when the mean is indicated. You may rather capture this as “The mean number of 33 (n=840) prescribed medications was 1.4 (SD=xyz)…”.

Response: The standard deviation has been included following the format suggested (Line 33)

**Response to Reviewer #5**

1. Comment: the study needs to be written in more technical language. it lacks consistency in tenses in sentences. the sample size of the prescriber is very less and analysis on prescription does not provide any new information.

Response: The manuscript has been edited to improve its technical clarity and grammatical consistency. Also, the number of prescribers interviewed reflects the total number of prescribers in that department during the period of the study. Finally, the inability to connect the prescriber interviews to the prescriptions analyzed has been stated as a limitation

Sincerely,

Nana Ama Buadiba Osei
